# Anti-fibrotic impact of Carvedilol in a CCl-4 model of liver fibrosis via serum microRNA-200a/SMAD7 enhancement to bridle TGF-β1/EMT track

**DOI:** 10.1038/s41598-018-32309-1

**Published:** 2018-09-25

**Authors:** Sara A. El-Wakeel, Rania M. Rahmo, Hanan S. El-Abhar

**Affiliations:** 1 Department of Pharmacology & Toxicology, Faculty of Pharmacy, Misr International University, Cairo, Egypt; 2 Department of Pharmacology & Toxicology, Faculty of Pharmacy, Cairo University, Cairo, Egypt

## Abstract

Circulating microRNAs (miRNAs) play a role in modulating the prevalence of fibrosis and have been a target of the cardiac anti-fibrotic effect of Carvedilol. However, the impact of miRNAs on the hepatoprotective effect of this non-selective β-blocker has not been yet elucidated. Hence, the current goal is to evaluate the potential role of circulating miR-200a in the hepatic anti-fibrotic pathway of Carvedilol. Male Wistar rats were randomized into normal, CCl_4_ (2 ml/kg, i.p, twice weekly for 8 weeks), and CCl_4_ + Carvedilol (10 mg/kg, p.o, daily). Carvedilol over-expressed the circulating miR-200a to modulate epithelial mesenchymal transition (EMT) markers (vimentin, E-Cadherin). In turn, Carvedilol increased SMAD7 gene expression and protein content to attenuate the pro-fibrogenic marker transforming growth factor β1 (TGF-β1) and the inflammatory markers (p-38 MAPK and *p*-S536-NF-κB p65). The anti-fibrotic potential was reflected on the decreased expression of the mesenchymal product and EMT marker α-SMA, besides the improved histopathological examination, and the fibrosis scores/collagen quantification to enhance liver functions (AST, ALT, ALP, and AST/platelet ratio index; APRI). In conclusion, circulating miR-200a/SMAD7/TGF-β1/EMT/MAPK axis is crucial in the hepatic anti-fibrotic mechanism of Carvedilol.

## Introduction

Hepatic fibrosis is a process defined by the distortion of hepatic parenchymal cells and their replacement by extracellular matrix or scar^1^. This process is reversible, however, in case of chronic damage it may progress to cirrhosis, a stage that may end up by hepatocellular carcinoma^2^. Hepatotoxins are among the common etiologies highlighting liver fibrosis, such as carbon tetrachloride (CCl_4_), which provokes liver injury that reaches fibrosis or cirrhosis according to the duration of damage^3^. Activation of hepatic stellate cells (HSCs) and their acquisition of a myofibroblastic phenotype expressing alpha smooth muscle actin (α-SMA), is a well-established event in the pathogenesis of liver fibrosis^4^. However, recent evidence implicated that epithelial mesenchymal transition (EMT), a process in which epithelial cells acquire a gradual loss of their epithelial phenotype and gain the characteristics of mesenchymal cells, is critical in liver fibrosis pathology, as well. Therefore, fibroblasts were found to be produced from epithelial cells through EMT and not only from HSCs^5^. EMT process involves the loss of the epithelial cell adhesion molecules, like E-Cadherin and Zona occludens (Zo-1) and their substitution with the mesenchymal markers: vimentin, fibronectin, collagen and matrix metalloproteinases (MMP-2 and MMP-9)^6,7^.

MicroRNAs (miRNas) are endogenous small (20–25) nucleotides non-coding RNAs targeting messenger RNA in the cytoplasm and leading to the inhibition of their transfer and translation, hence regulating protein expression involved in fibrosis, inflammation, and oncogenesis. Alteration of circulating miR200a in the serum reveals pathological conditions of the liver and may function as a non-invasive diagnostic marker in progressive liver diseases^8^. It has been reported that miR200 family regulates transforming growth factor (TGF-β1)-induced renal tubular EMT through the SMAD pathway^9^. SMADs are a family of proteins that modulate the fibrogenic marker TGF-β1 signaling, which is known to wane the epithelial cell adhesion molecule E-Cadherin, and to increase vimentin, a key marker for EMT, and this process was enhanced by the deletion of SMAD7^10,11^.

SMAD7 protein is known as the inhibitory SMAD (I-SMAD) or the protective SMAD that switches off signaling of TGF-β1/SMAD, as well as nuclear factor kappa (NF-κB) in liver fibrosis. The latter transcription factor, as well as mitogen activated protein kinase (p38 MAPK) are major cues involved in SMADs signaling^11–13^.

Supporting the notion that liver fibrosis is a reversible process, the strategy of emerging anti-fibrotic drugs has been advanced^14^. In 2009, Porter and Turner^15^ clarified the correlation between β-blockers and fibrosis; the authors associated the activation of cardiac myofibroblasts to several factors, including hormones as norepinephrine (NE) and highlighted the crucial role of the increased levels of these factors in heart remodeling. Hence, they suggested that certain cardiovascular drugs, including β-blockers, can reduce myocardial remodeling specifically *via* modulatory effects on cardiac fibroblasts^15^. Apart from its antihypertensive effect, Carvedilol, was proven for its anti-fibrotic activity^16–18^.

A recent study has associated the myocardial anti-fibrotic role of Carvedilol to a subtype of miRs and SMADs^19^. However, in the liver, the anti-fibrotic mechanism of Carvedilol was explained in terms of its antioxidant and anti-inflammatory properties^16,18,20,21^, yet the exact signaling pathways underlying its hepatic anti-fibrotic activity in the context of miR-200a, SMAD7 and EMT are still unknown, which is the aim of this work.

In this study, miR-200a/SMAD7 stream is investigated as one of the pathways contributing to the hepato-protective mechanism of Carvedilol through the suppression of EMT.

## Results

### Effect of Carvedilol on miR-200a and SMAD7 in CCl_4_-induced liver fibrosis

**Figure 1:**
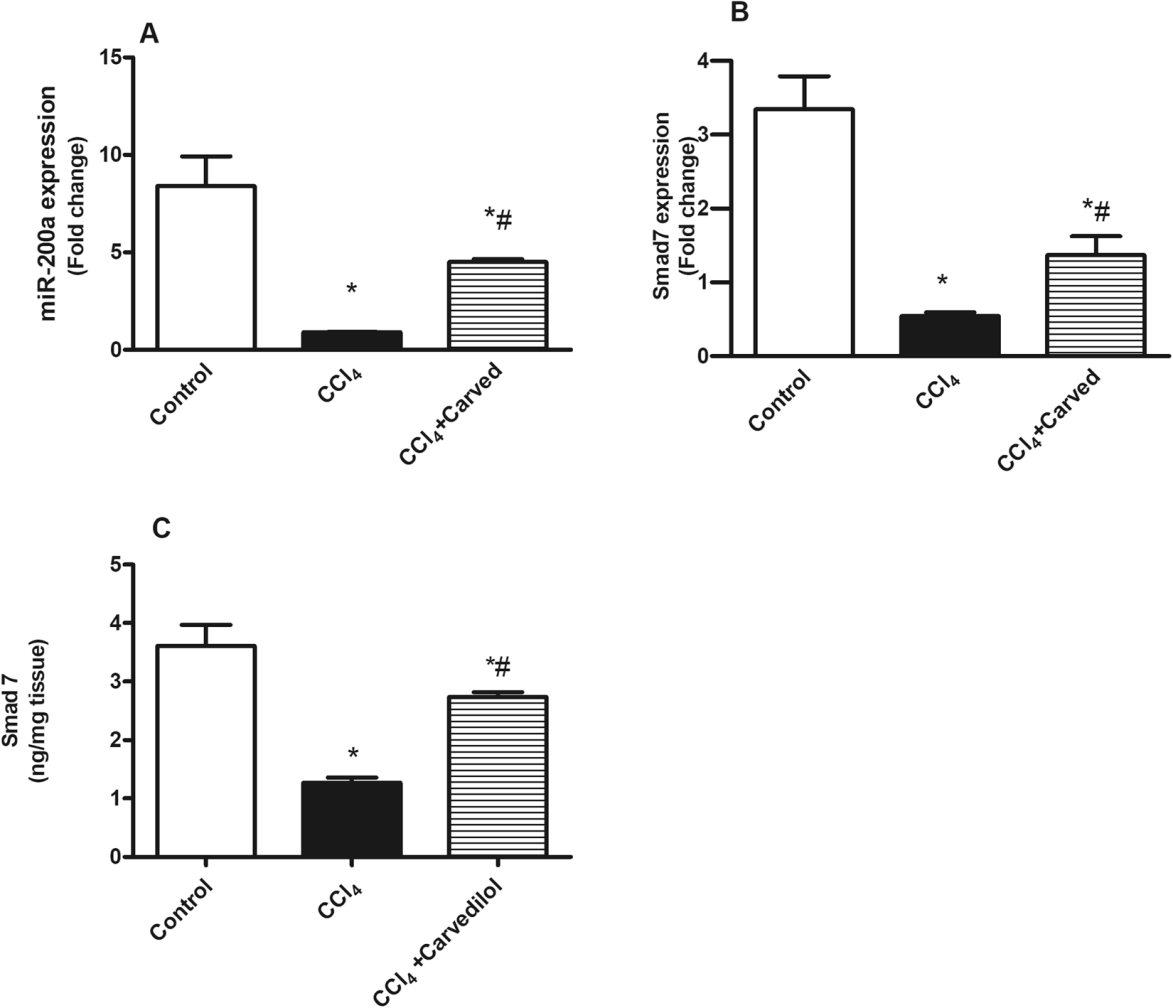
Effect of Carved on gene expression of (**A**) serum miR 200a, and (**B** & **C**) hepatic gene expression/protein content of SMAD7. Values are expressed as mean ± S.D (n = 7). Statistical analysis was carried out using one-way ANOVA followed by Tukey’s *post hoc* test, *P* < 0.05. As compared to (*) control group and (#) CCl_4_ intoxicated group. Carved treatment (10 mg/kg, p.o, daily) started 2 weeks post CCl_4_ (2 ml/kg, i.p) initiation until week 8. Carved: Carvedilol; CCl_4_: carbon tetrachloride.

As depicted in Fig. 1, CCl_4_ intoxicated group showed a marked downregulation of (A) serum miR-200a gene expression, as compared to the control group. This effect entailed its downstream SMAD7, where the insult decreased its hepatic (B) gene expression and (C) protein content. On the other hand, Carvedilol upregulated serum miR-200a and hepatic SMAD7 by 5 and 2.5 fold, respectively, this effect was reflected also on the protein content of SMAD7 (2.1 folds).

### Effect of Carvedilol on hepatic content of mesenchymal to epithelial transition (MET)/epithelial to mesenchymal transition (EMT) markers in CCl_4_-induced liver fibrosis

**Figure 2:**
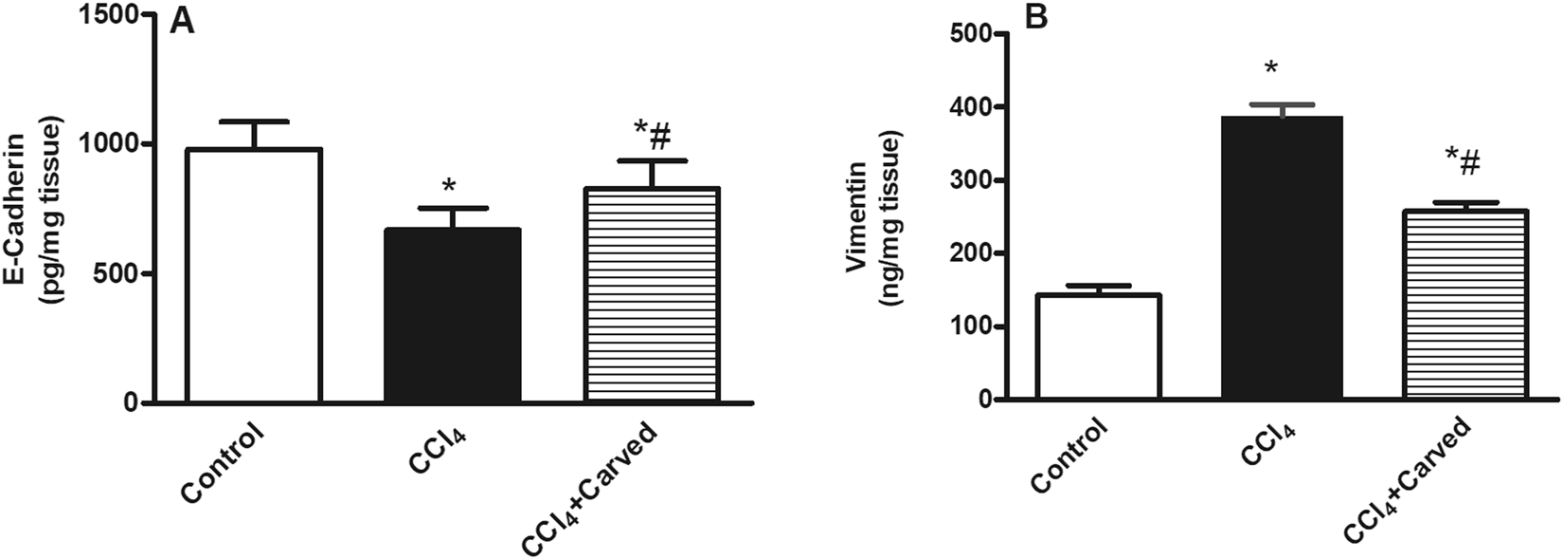
Effect of Carved on hepatic contents of (**A**) E-Cadherin and (**B**) vimentin. Values are expressed as mean ± S.D (n = 7). Statistical analysis was carried out using one-way ANOVA followed by Tukey’s *post hoc* test, *P* < 0.05. As compared to (*) control group or (#) CCl_4_ intoxicated group. Carved treatment (10 mg/kg, p.o, daily) started 2 weeks post CCl_4_ (2 ml/kg, i.p) initiation until week 8. Carved: Carvedilol; CCl_4_: carbon tetrachloride.

In Fig. 2, CCl_4_ reduced the hepatic content of (A) E-Cadherin (668.7+/− 84.67 *vs* normal 979.1+/−106.6), while increased that of (B) vimentin to reach 2.7 folds, as compared to the vehicle group. Carvedilol, however, reverted the CCl_4_ effects to raise the MET marker E-Cadherin significantly and to reduce the EMT marker vimentin.

### Effect of Carvedilol on hepatic content of inflammatory markers in CCl_4_-induced liver fibrosis

**Figure 3:**
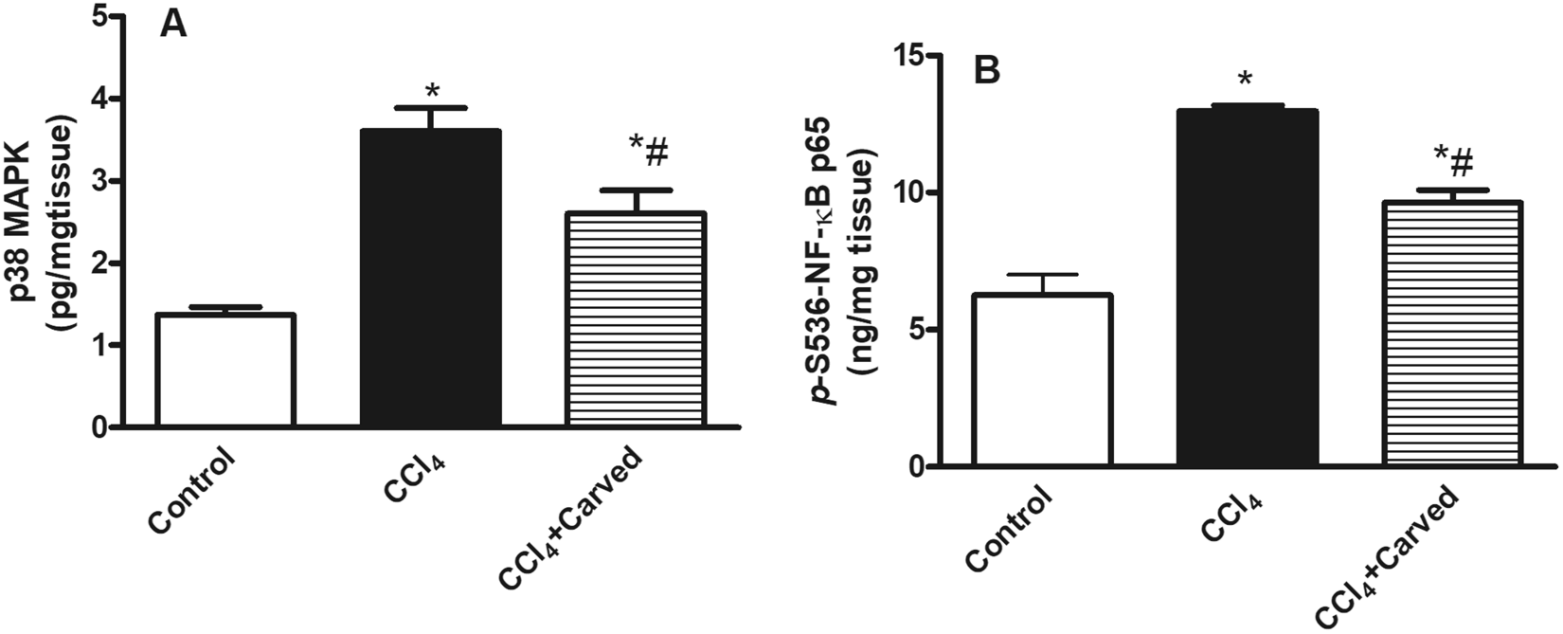
Effect of Carved on hepatic contents of (**A**) p38 MAPK and (**B**) *p*-(S536)NF-κB p65. Values are expressed as mean ± S.D (n = 7). Statistical analysis was carried out using one way ANOVA followed by Tukey’s post hoc test, P < 0.05. As compared to (*) control group or (#) CCl_4_ intoxicated group. Carved treatment (10 mg/kg, p.o, daily) started 2 weeks post CCl_4_ (2 ml/kg, i.p) initiation until week 8. Carved: Carvedilol; CCl_4_: carbon tetrachloride.

The insulted group (Fig. 3) showed a significant increase in the hepatic contents of (A) p38 MAPK and (B) the *p*(S536) NF-κB p65 by 2.7 and 2.1 folds, respectively as compared to the vehicle group. However, Carvedilol counteracted these increments by reducing their levels, respectively by 28% and 26%, as compared to the insulted group.

### Effect of Carvedilol on liver function tests in CCl_4_-induced liver fibrosis

**Figure 4:**
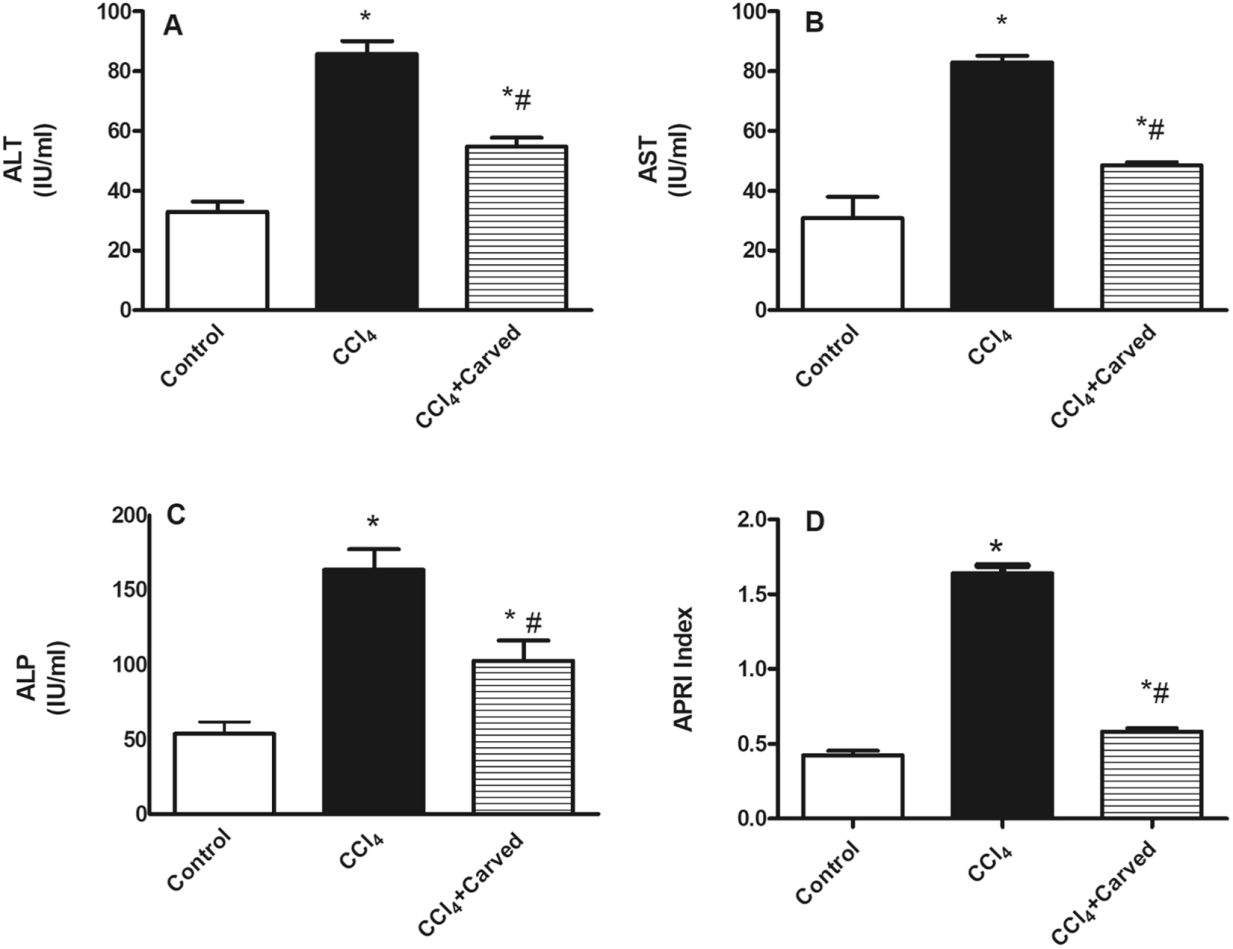
Effect of Carved on liver function tests (**A**) ALT, (**B**) AST, (**C**) ALP and (**D**) APRI. Values are expressed as mean ± S.D (n = 7). Statistical analysis was carried out using one way ANOVA followed by Tukey’s *post hoc* test, *P* < 0.05. As compared to (*) control group or (#) CCl_4_ intoxicated group. Carved treatment (10 mg/kg, p.o, daily) started 2 weeks post CCl_4_ (2 ml/kg, i.p) initiation until week 8. ALP: alkaline phosphatase; ALT: alanine transaminase; AST: aspartate transaminase; APRI: AST/platelet ratio index; Carved: Carvedilol; CCl_4_: carbon tetrachloride.

Figure 4 confirmed the CCl_4_-induced liver injury, where in the intoxicated group, the serum levels of (A) ALT, (B) AST, and (C) ALP were elevated by 2.5, 2.7, 3.1 folds, respectively, as compared to the vehicle group, while treatment with Carvedilol has reduced these values significantly. Moreover, (D) the AST to platelet ratio index (APRI) has increased in the insulted group by 3.9 folds relative to the vehicle group, whereas Carvedilol reduced it by 69%, as compared to the CCl_4_ intoxicated group.

### Effect of Carvedilol on immune-histochemical examination of pro-fibrogenic marker/HSC activation markers

**Figure 5:**
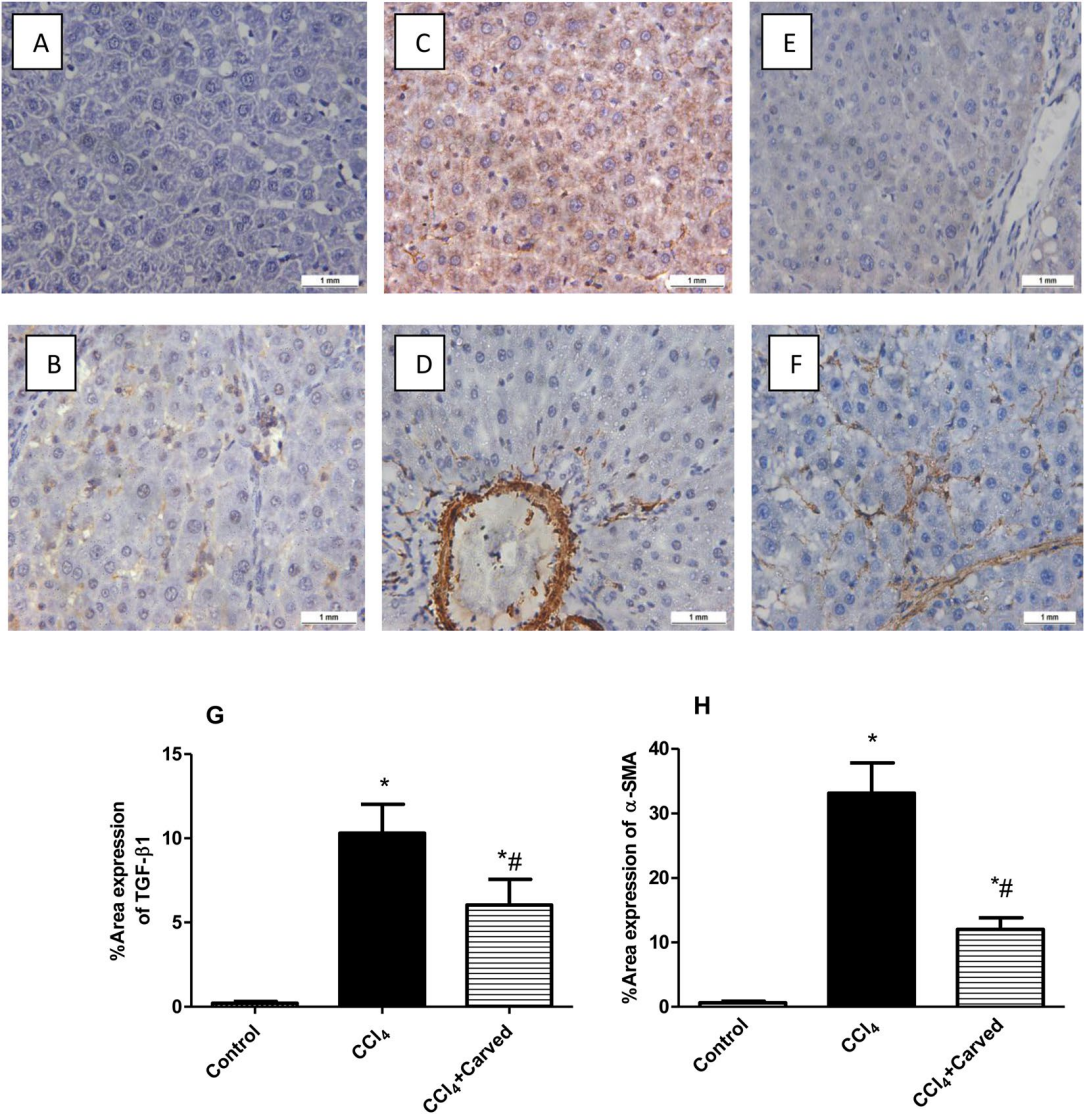
Effect of Carved on immunohistochemical examination of TGF-β1 and α-SMA and their percentage area expression. The immunostained liver cells of normal sections show normal architecture with negative immune-reactivity for (**A**) TGF-β1 and (**B**) α-SMA antibodies. Oppositely, sections from CCl_4_ intoxicated group show positive immunoreactivity for (**C**) TGF-β1 and (**D**) α-SMA antibodies (brown discoloration), whereas Carvedilol decreased the immune-reactivity for (**E**) TGF-β1 and (**F**) α-SMA (x100). The percentage area expression of TGF-β1 and α-SMA are shown in panels G and H, respectively. Values are expressed as mean (3 sections/rats/group) ± S.D. Statistical analysis was carried out using one-way ANOVA followed by Tukey’s *post hoc* test, *P* < 0.05. As compared to (*) control group or (#) CCL_4_ intoxicated group. Carved treatment (10 mg/kg, p.o, daily) started 2 weeks post CCl_4_ (2 ml/kg, i.p) initiation until week 8. Carved: Carvedilol; CCl_4_: carbon tetrachloride; TGF-β1: transforming growth factor β1; α-SMA: alpha smooth muscle actin.

As depicted in Fig. 5a, immune-histochemical staining analysis showed that Carvedilol has reduced hepatic expression of the pro-fibrogenic marker TGF-β1 and the marker of HSC activation α-SMA showing minimal brown staining, as compared to the CCl_4_ intoxicated group. These results were further confirmed by the percentage area expression of TGF-β1 and α-SMA, as shown in Fig. 5b and c, respectively.

### Effect of Carvedilol on liver histopathological changes

**Figure 6:**
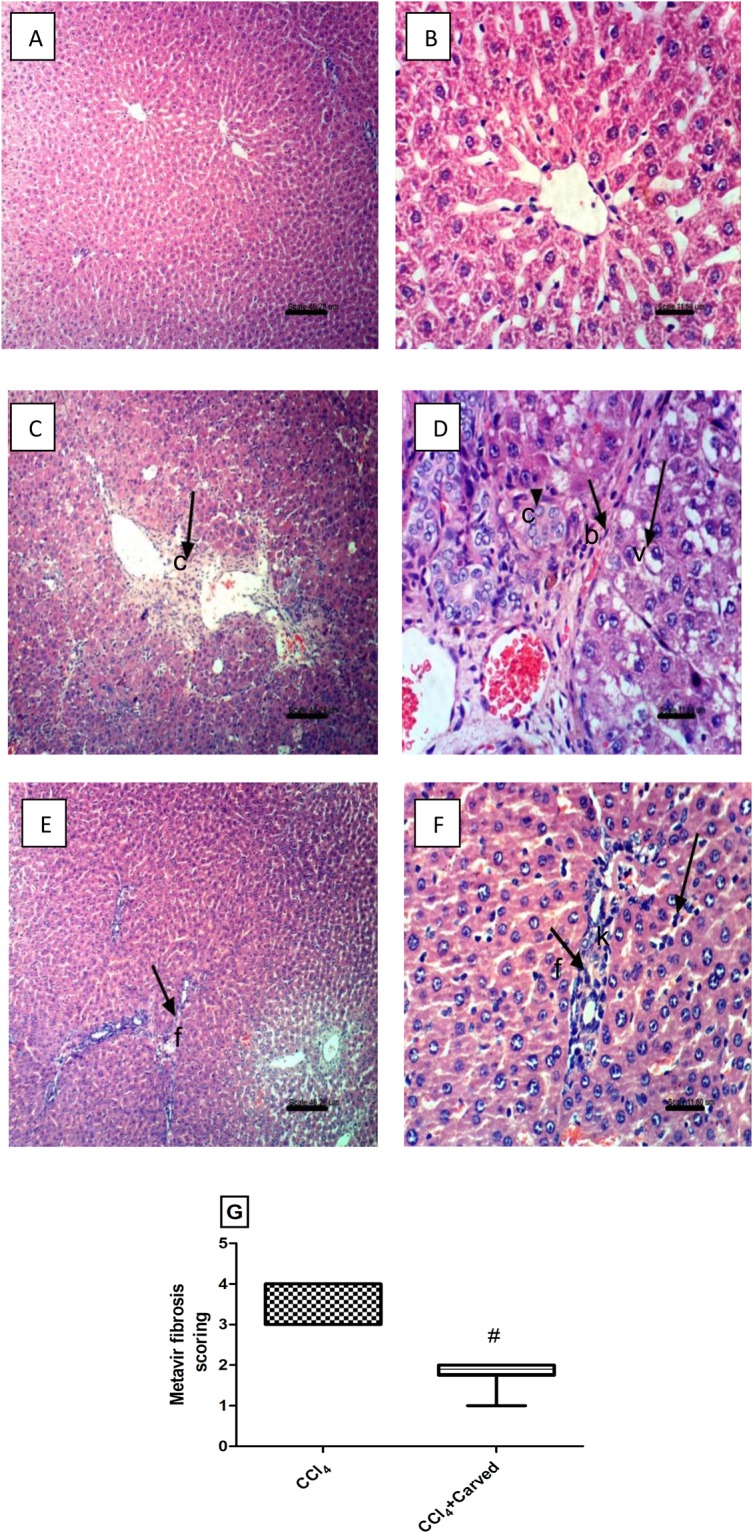
Effect of Carved on photomicrographs of liver sections stained with H&E. Sections of (**C,D**) CCl_4_ intoxicated group show collagen fibers deposition in the portal triad [c], vacuolar degeneration of hepatocytes [v], appearance of newly formed bile ductuoles [b] (arrows), as compared to those of (**A,B**) normal group. Sections of (**E,F**) Carved treated group show fine strands of fibroblasts proliferation and instead to prevent repitition [f], Kupffer cells activation and few leucocytes in the portal triad [k] (arrows). (x100; scale bar = 46.2μm; x400; scale bar = 11.6 μm). Panel G reveals METAVIR Fibrosis scoring. Data are expressed as median values (max-min). Statistical analysis was carried out using non-parametric Mann Whitney U test between CCl_4_ and CCl_4_ + Carved (#), P < 0.05. Carved treatment (10 mg/kg, p.o, daily) started 2 weeks post CCl_4_ (2 ml/kg, i.p) initiation until week 8. Fibrosis score was evaluated in ten randomly selected fields from each slide. Carved: Carvedilol; CCl_4_: carbon tetrachloride.

Liver photomicrographs (H & E) (Fig. 6) reveal that sections of (C & D) CCl_4_ intoxicated group show deposition of collagen fibers in the portal triad [c], vacuolar degeneration of hepatocytes [v], and newly formed bile ductuoles [b], as compared to the (A & B) normal intact histological structure of hepatocytes seen in the vehicle treated sections. Sections of (E & F) Carvedilol treated group reveal improvement in liver structure, where the sections show fine strands of fibroblasts proliferation in the portal triad [f], mild activated Kupffer cells in the portal triad [k], as compared to the CCl_4_ intoxicated group. These changes are summarized in panel G according to the Metavir fibrosis scoring.

### Effect of Carvedilol on collagen deposition in liver photomicrographs stained with Masson-Trichome stain

**Figure 7:**
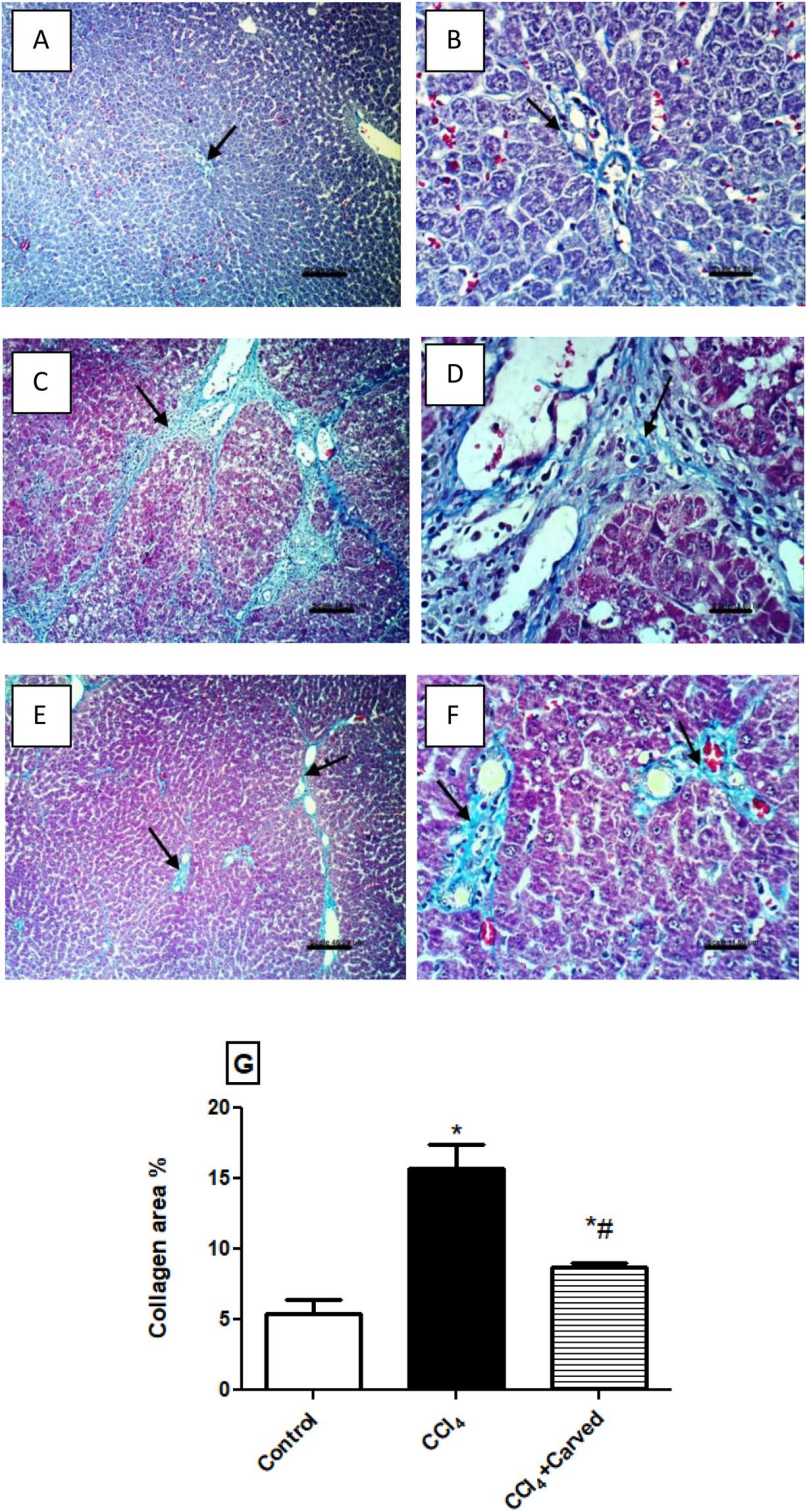
Effect of Carved on photomicrographs of liver tissues stained with Masson Trichome stain. Sections of (**C,D**) CCl_4_ intoxicated group show extensive fibrosis and strong histochemical reaction for collagen fibers in the portal triad and around the hepatic lobules (arrows), relative to sections of (**A,B**) normal group showing no histochemical reaction for collagen fibers. Sections of animals treated with (**E,F**) Carved show a weak histochemical reaction for collagen fibers (arrows) (x100, scale bar = 46.2μm; x400: scale bar = 11.6 μm). Panel (**G**) represents percentage area of collagen content in liver tissues. Values are expressed as mean ± S.D (n = 3sections/rat/group). Statistical analysis was carried out using one way ANOVA followed by Tukey’s *post hoc* test, *P* < 0.05. As compared to (*) control group or (#) CCl_4_ intoxicated group. Carved treatment (10 mg/kg, p.o, daily) started 2 weeks post CCl_4_ (2 ml/kg, i.p) initiation until week 8. Carved: Carvedilol; CCl_4_: carbon tetrachloride.

In Fig. 7, MTC stain also supported the H & E sections; it shows no histochemical reaction for collagen fibers in the (A & B) normal control group, while sections of (C & D) CCl_4_ intoxicated group reveal extensive fibrosis and strong histochemical reaction for collagen fibers in the portal triad and around the hepatic lobules (arrows). On the other hand, sections of (E & F) Carvedilol treated group show weak histochemical reaction of collagen fibers, as compared to the CCl_4_ intoxicated group. Panel G shows the changes in area percentage of collagen.

**Figure 8:**
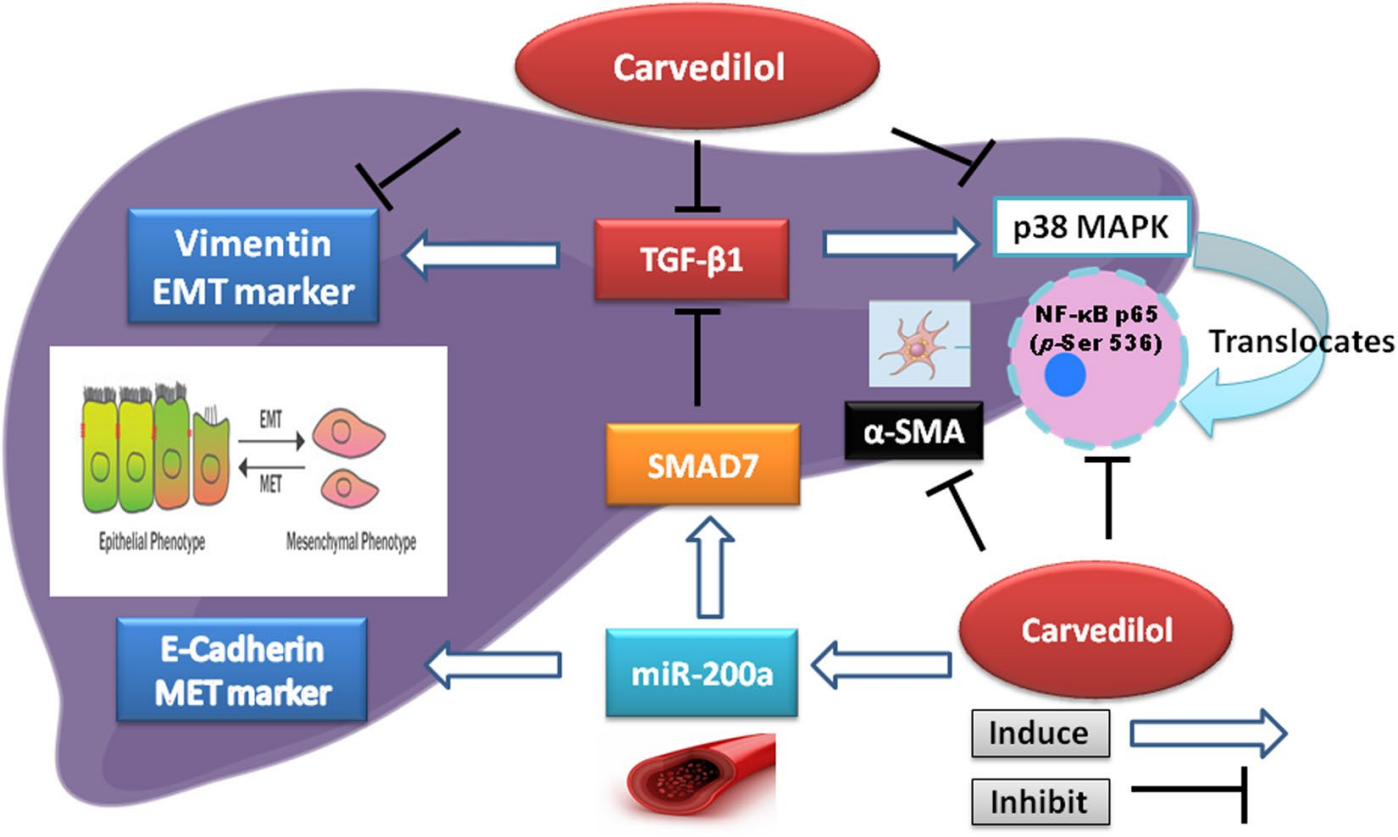
A graphical abstract highlighting the Carvedilol potential mechanism via miR200-a targeting SMAD7/TGF-β1/EMT/MAPK. Carvedilol enhances the gene expression of the circulating anti-fibrotic miR-200a. MiR-200a stimulates the prototypical epithelial marker E-Cadherin, as well as the protective SMAD7, which in return hinders the action of the pro-fibrogenic cytokine TGF-β1. Carvedilol inhibits TGF-β1, the EMT marker α-SMA, *p*-(ser536)NF-κBp65/MAPK and the mesenchymal marker vimentin. MiR-200: microRNA-200a; TGF-β1: transforming growth factor beta 1; EMT: epithelial mesenchymal transition; *p*-(ser536)NF-κBp65: phosphorylated nuclear factor kappa b at serine 536; MAPK: mitogen activated protein kinase.

A sum-up of the Carvedilol hepatic anti-fibrotic mechanismsTo recapitulate the current tackled mechanisms/pathways by Carvedilol, Fig. 8 shows that CCl4 abated miR200-a/SMAD7, as well as E-Cadherin, while enhanced the fibrotic-related markers (vimentin, TGF-β1, α-SMA), as well as the inflammatory markers (p38MAPK, *p*-NF-κBp65) to further augment liver injury. Administration of Carved, however, proved its beneficial effect, as it modulated these pathways significantly in favor of reducing fibrosis.

## Discussion

Liver fibrosis is described as a response to chronic injury that can progress to organ failure upon extended damage^22^. This ailment is an endpoint of several factors that assimilate to activate the fibrogenic process. Among these factors is the activity of the sympathetic nervous system, which regulates hepatic fibrogenesis by exerting an effect on hepatic stellate cells^23^. Moreover, Nuamnaichati *et al*. recently described the mechanism of β-blockers in fibrosis revealing that continuous stimulation of the β-adrenergic receptors induces the synthesis and secretion of growth factors in cardiac myocytes that activate cardiac fibroblast, thus, imposing a correlation between these receptors and the activation of fibroblast^24^.

Despite this ailment is a historic disease, yet anti-fibrotic therapies are powerless to restrain liver fibrosis in an efficient approach^25^. Nevertheless, Carvedilol, a non-selective β-blocker approved for the treatment of hypertension, has been proven for its efficient hepatic anti-fibrotic properties that are mainly ascribed to its antioxidant and anti-inflammatory properties^16,18,21^. However, in our study, we have evaluated the impact of circulating miR-200a/SMAD7/TGF-β1 axis and the suppression of EMT on the anti-fibrotic effect of Carvedilol.

In this study, Carvedilol significantly upregulated the serum miR-200a gene expression, as compared to the CCl_4_ intoxicated group, an effect that is partly responsible for the decreased hepatic fibrosis. In support to our findings, previous studies reported that the expression of miR-200a is downregulated in case of liver fibrosis^26^.

Moreover, growing evidence showed that miRNAs participated in hepatic fibrosis through targeting SMAD proteins in liver^27^. SMADs are a group of proteins that mediate the TGF-β signaling and they are targeted by miRNAs^11^. TGF-β is considered as the most powerful commanding pro-fibrogenic cytokine that triggers fibrosis through the enhancement of SMAD-based pathways^28^. This association was further reinforced by a research article revealing that the over expression of miR-200a inhibited SMAD3 activity and attenuated TGF-β1-induced fibrosis^29^; however, the correlation between miR-200a and SMAD7 in liver has not been studied before.

In the current study and in parallel to the miR-200a results, hepatic level of SMAD7 was upregulated in the Carvedilol treated group, as compared to the CCl_4_ intoxicated one. This effect was associated with a decrease in the protein expression of TGF-β1, as documented by the current immune-histochemical examination. Our results concur with an earlier study in a model of renal fibrosis^9^, where Xiong *et al*. established a correlation between SMAD7 and miR-200 family and they stated that activation of SMAD7 leads finally to hindrance of the TGF-β1-mediated miR-200 downregualtion. Furthermore, a recent study conducted on cardiac fibrosis model, established in human aortic endothelial cells, reported that increased TGF-β1 is responsible for the downregulated expression of miR-200a^30^.

A further confirmation for the Carvedilol anti-fibrotic capacity, immunostaining of liver sections with α-SMA antibody divulged a minimal staining of liver tissues in Carvedilol treated group indicating a decline in collagen content, as compared to the CCl_4_ intoxicated group, while the latter group showed a brown staining as compared to the vehicle treated group. Similarly, an earlier study confirmed the α-SMA immune-histochemical results^16^. The increased protein expression of α-SMA can be owed to the enhanced TGF-β1, in the current model to match previous findings; Li *et al*.^31^ have reported that increased TGF-β1 triggers a rise in α-SMA in a model of renal fibrosis.

EMT is a basic part of liver fibrosis pathology^32^; E-Cadherin and vimentin are classified as type 2 epithelial mesenchymal transition (EMT) markers and this type is responsible for organ fibrosis, wound healing, and tissue regeneration in the liver^33^. In tissue fibrosis, the alteration in E-Cadherin during EMT process is the archetype epithelial marker^34,35^. However, vimentin is a mesenchymal product recognized as type III intermediate filament protein, expressed at the site of injury during the repair phase and declines subsequent to injury resolution^5^. Indeed, E-Cadherin downregulation is mediated through vimentin upregulation to hinder E-cadherin trafficking to the cell^36^.The beneficial effect of Carvedilol on miR-200a entailed the modulation of EMT markers, verified by decreasing vimentin and increasing E-Cadherin, as compared to the CCl_4_ intoxication. Our findings were in line with Lu *et al*., who revealed that miR-200a hinders EMT *via* the inhibition of vimentin in a pancreatic cancer model^37^. Moreover, parallel studies carried in a model of kidney fibrosis proved that the expression of miR-200 family obstructed EMT and maintained a high level of E-Cadherin^9^. Additionally, a recent study further supports our results; the authors mentioned in a model of pancreatic fibrosis that TGF-β1 promotes EMT, while miR-200a inhibits it *via* a negative regulation of TGF-β1-induced pancreatic stellate cells activation^38^. It is noteworthy to mention that Gong *et al*. also proved in a recent model of proximal tubules EMT that miR-200a impeded TGF-β1-induced EMT^39^. The reduction of vimentin and the increase of E-Cadherin were enhanced by the upregulation of SMAD7 and the inhibition of TGF-β1.

CCl_4_ is a toxin recognized for the induction of liver damage, partially *via* the production of inflammatory cytokines; these cytokines are associated with the activation of mitogen-activated protein kinases (MAPKs) and NF-κB^40^. In normal cells, NF-κB dimer interact with IκBα and produce an inactive complex localized in the cytoplasm. Under stressful conditions, the hepatic cells are activated and this leads to the phosphorylation/degradation of IκBα, which frees activated NF-κB to be translocated into the nucleus^41^. Liver fibrosis pathogenesis is linked to p38 MAPK that phosphorylates and activates the nuclear kinase mitogen- and stress-activated protein kinase (MSK1). In return, activation of MSK1 plays a central role in the activation of NF-κB^42^. Activated p38MAPK can be the missing loop between fibrosis and inflammation, where apart from its fibrogenic role, increased TGF-β1 extends its effect to activate p38MAPK, which in turn stimulates the transcription factor NF-κB. In 2008, Sorrentino *et al*. correlated p38MAPK with the fibrogenic cytokine TGF-β1 after its binding to the TBR1 receptor in a SMAD-independent pathway^43^. This fact should not rule out the role of SMAD7, which when downregulated activates NF-κB p65 through inhibiting IκBα proteins expression to endorse its degradation by phosphorylation^11,44^. These evidences are in agreement with our results, where the hepatic levels of p38MAPK and the active form NF-κB p65 (*p*Ser 536) were significantly raised in CCl_4_ intoxicated group, as compared to the normal control group, whereas treatment with Carvedilol significantly diminished them compared to the intoxicated group. We may conclude that the anti-inflammatory capacity of Carvedilol relies on both the inhibition of TGF-β1, as well as the overexpression of SMAD7 to coincide with the results of Wang *et al*. in a renal fibrosis model^44^.

The Carvedilol anti-fibrotic effect was mirrored on the improved liver function; as a consequence to the CCl_4_-induced hepatic injury, liver enzymes; *viz*., AST, ALT and ALP, were markedly elevated, whereas treatment with Carvedilol leveled them off to signify its hepato-protective effect and to match with a previous study^16^. The ratio of AST to platelet count (APRI) is one of the non-invasive methods used in the assessment and diagnosis of liver fibrosis^45^. In our study, CCl_4_ intoxicated group revealed a rise in APRI, however the Carvedilol treated group showed a significant decline in APRI, as compared to the CCl_4_ group. Additionally, our results were associated by an improvement in the histopathological examination using H&E and MTC stain, besides the morphometrical collagen quantification test. These examinations revealed collagen deposition and extensive fibrosis detected as karyomegaly of hepatic nuclei and hyperplasia of bile duct in the CCl_4_ intoxicated group and this was in agreement with Li *et al*.^46^. On the other hand, Carvedilol improved liver histology and hindered collagen deposition triggered by CCl_4_. The significant differences between the intoxicated and Carvedilol groups in fibrosis scoring confirmed the results of the histopathology and morphometry, as Carvedilol has reduced the semi-quantitative fibrosis score in the liver specimens^47^. Our histopathological and morphometrical analysis were in parallel with previous literature^16^.

Our results established a recent approach associating the anti-fibrotic mechanism of the non-selective β-blocker Carvedilol with the circulating miR-200a, the newly diagnostic non-invasive biomarkers of liver fibrosis, SMAD7, and through the suppression of EMT.

## Materials and Methods

### Animals

Thirty adult male Wistar rats (180–200 g) were purchased from National Research Center (Cairo, Egypt). Animals were kept under controlled environmental conditions at a constant temperature (23 ± 2 °C), humidity (60 ± 10%) and a light/dark (12/12 h) cycle at the animal facility of Faculty of Pharmacy, Misr International University (MIU). Food (standard diet pellets; EL-Nasr Co., Abu Zaabal, Egypt) and water were available *ad libitum* until the beginning of the experiment. Animal handling and experimental protocols comply with the Guide for the Care and Use of Laboratory Animals^48^, and were approved (PT:1772) by the Research Ethical Committee of the Faculty of Pharmacy, Cairo University (Cairo, Egypt).

### Induction of fibrosis and experimental design

Rats were randomly distributed into three groups (10 rats each); animals in group1 received the vehicle dimethyl sulphoxide (1% DMSO), whereas those in group 2 received CCl_4_ in olive oil (1:1 v/v) (El Gomhorya Co, Cairo, Egypt; 2 ml/kg; i.p) twice weekly for 8 weeks^49^. Rats in group 3, received CCl_4_ as in group 2 and were gavaged Carvedilol (Carvid^®^ MAP S.A.E, Cairo, Egypt) in a dose of 10 mg/kg. Two weeks after initiation of CCl_4_, Carvedilol (suspended in 1% DMSO) was administered daily till week 8^16^.

Following the termination of experimental protocol, rats were anesthetized with an overdose of thiopental and blood samples were withdrawn from the femoral vein. One aliquot was immediately analyzed manually for platelet count and then confirmed using hemocytometer to be used later for calculating the AST platelet ratio index. Sera were separated from another aliquot and were used for the assessment of liver function tests and circulatory miR-200a. The animals were sacrificed and the liver was harvested and divided into 3 portions. One portion was homogenized in phosphate buffered saline (PBS; 10% w/v), divided into aliquots, and frozen at −80 °C until analysis.

### Hepatic content of SMAD7, vimentin, E-Cadherin, p38MAPK and p-S536-NF-κB p65

The rat sandwich ELISA kits were purchased from Life Span BioSciences (Seattle, USA) to assess SMAD7 (cat#LS-F2280) and from MyBiosource (Vancouver, Canada) to assess Vimentin (cat#S032693). Moreover, ELISA kits for E-Cadherin (cat#ab202413), p38MAPK (cat#ER1138), and *p*-S536-NF-κBp65 (cat#PEL-NFKBP65-S536) were procured from Abcam’s ELISA kit (Cambrige, UK), Fine test (Wuhan, Hubei, China), and Ray Biotech (Georgia, USA), respectively. These measurements were performed according to the manufacturer’s instructions.

### Assessment of hepatic function

Serum aminotransferases (alanine transaminase; ALT, aspartate transaminase; AST) and alkaline phosphatase (ALP) were assessed using the corresponding Stanbio colorimetric assay kits (Liqui-UV; TX, USA).

### Histopathological and immune-histochemical examination

Specimens from 3 representative animals/group were fixed in 10% buffered formaldehyde, embedded in paraffin, and sliced with a microtome into 5μm sections. These sections were used for the histopathological and immuno-histochemical examinations. Liver sections were deparaffinised in xylene, washed with ethanol, and placed on glass slides to be stained with Hematoxylin and Eosin (H&E) and Masson trichome (MTC) for histopathological examination of structural changes.

The remaining slices were then immuno-stained with primary antibody anti-TGF-β1 or anti-α-SMA antibody (Santa-Cruz Biotechnology, CA, USA) at a concentration of 1 μg/ml containing 5% BSA (bovine serum albumin) in buffered saline, then incubated with goat anti-rabbit secondary antibody. The percentage expression area of TGF-β1 and α-SMA, as well as liver sections were examined under a light microscope using Leica Quin Plus version 3 (magnification power were x100 x400).

### Morphometric analysis and fibrosis scoring

Morphometric analysis of fibrosis was quantified as the mean of 3 consecutive sections/rat/group as a percentage of the total area that was positive for MTC stain in the digital photomicrographs using a computerized image analysis system (Leica Quin plus version 3 software/magnification power x400). The extent of liver fibrosis was evaluated blindly by a pathologist based on METAVIR liver fibrosis scoring system^47^. The scale ranges from 0–4; F0: No fibrosis, F1: Portal fibrosis without septa, F2: Portal fibrosis with few septa, F3: Numerous septa without cirrhosis, and F4: Cirrhosis.

### Quantitative real-time PCR for hepatic SMAD7 and serum miR-200a

Total RNA was extracted and purified from serum samples or homogenized liver tissue samples using using miRneasy mini kit including QIAzol Lysis Reagent according to the manufacturer’s instructions (Qiagen; Hilden, Germany). For the serum samples, this protocol allows the purification of separate fractions enriched in miR and other small RNA species; for miR recovery a RNeasy MinElute clean up kit was used according to the manufacturer’s instructions. SMAD7 and MiR200a gene expression were carried out using gene-specific oligonucleotide primers (Invitrogen, Thermo Fisher Scientific, Inc; CA, USA). Primers sequence for both genes and the housekeeping gene β-actin are presented in Table 1. Total RNA was reverse transcribed to cDNA and amplified in one tube using one-step RT-PCR with SYBR^**®**^Green iScript PCR kit (BioRad, CA, USA). Complete reaction mix was incubated in a real time thermal detection cycler as follows: cDNA synthesis at 50 °C for 10 min, reverse transcriptase inactivation 5 min at 95 °C followed by 30 to 45 cycles of amplification 10 s at 95 °C and 30 s at 55 °C to 60 °C. SMAD7 gene expression was carried out using gene-specific oligonucleotide primers (Invitrogen, Thermo Fisher Scientific, Inc; CA, USA). Mean Ct values were used to calculate the relative expression levels of the target gene for the experimental groups, relative to those in the control group. The gene expression data were normalized relative to the housekeeping gene β-actin using the 2^**−**ΔΔCt^ formula.

**Table 1:** Primers sequence of target genes.

Gene	Forward primer	Reverse primer
miR200a	5′CCTCTGTGGGCATCTTACCG-3′	5′TGGGTCACCTTTGAACATCGT-3′
SMAD7	5′-ACGACTTTTCTCCTCGCCTC-3′	5′TGGACAGTCTGCAGTTGGTTT-3′
β-Actin	5′-GCAGGAGTACGATGAGTCCG-3′	5′ACGCAGCTCAGTAACAGTCC-3′

### Statistical analysis

Parametric data are presented as mean ± S.D (n = 7), all statistical analyses were performed using one-way analysis of variance (ANOVA; *p* < 0.05) followed by Tukey’s multiple comparison *post hoc* test. Non-parametric data are presented as box and whiskers with median (max-min) and analysed by Mann-Whitney U test between model and treatment (GraphPad (Prism) software, version 5^®^).
